# 

**Published:** 1971-07

**Authors:** 


					W/
3f\32 3
-receive
BRISTOL
MEDICO-
CHIRURGICAL
JOURNAL
?*.C> l.>g
JULY 1971
VOL. 86 (iii) No. 319

				

